# Complete Mitochondrial Genome Assembly of an Upland Wild Rice Species, *Oryza granulata* and Comparative Mitochondrial Genomic Analyses of the Genus *Oryza*

**DOI:** 10.3390/life13112114

**Published:** 2023-10-25

**Authors:** Fen Zhang, Haiqi Kang, Lizhi Gao

**Affiliations:** 1 College of Agriculture, South China Agricultural University, Guangzhou 510642, China; zhangfen@stu.scau.edu.cn; 2 Tropical Biodiversity and Genomics Research Center, Engineering Research Center for Selecting and Breeding New Tropical Crop Varieties, Ministry of Education, Hainan University, Haikou 570228, China; kanghq@126.com

**Keywords:** mitochondrial genome, *Oryza granulata*, repeat sequences, horizontal transfer, phylogenetic, rearrangement

## Abstract

Wild upland rice species, including *Oryza granulata*, possess unique characteristics that distinguish them from other *Oryza* species. For instance, *O. granulata* characteristically has a GG genome and is accordingly classified as a basal lineage of the genus *Oryza*. Here, we deployed a versatile hybrid approach by integrating Illumina and PacBio sequencing data to generate a high-quality mitochondrial genome (mitogenome) assembly for *O. granulata*. The mitogenome of *O. granulata* was 509,311 base pairs (bp) with sixty-seven genes comprising two circular chromosomes, five ribosomal RNA (rRNA) coding genes, twenty-five transfer RNA (tRNA) coding genes, and thirty-seven genes coding for proteins. We identified a total of 378 simple sequence repeats (SSRs). The genome also contained 643 pairs of dispersed repeats comprising 340 palindromic and 303 forward. In the *O. granulata* mitogenome, the length of 57 homologous fragments in the chloroplast genome occupied 5.96% of the mitogenome length. Collinearity analysis of three *Oryza* mitogenomes revealed high structural variability and frequent rearrangements. Phylogenetic analysis showed that, compared to other related genera, *O. granulata* had the closest genetic relationship with mitogenomes reported for all members of *Oryza,* and occupies a position at the base of the *Oryza* phylogeny. Comparative analysis of complete mitochondrial genome assemblies for *Oryza* species revealed high levels of mitogenomic diversity, providing a foundation for future conservation and utilization of wild rice biodiversity.

## 1. Introduction

Of the 27 species recognized in the genus *Oryza*, 25/27 are wild species, while 2/27 represent the cultivated species [1,2,3]. Depending on morphological, cytological, and molecular features, members within the genus *Oryza* are sub-classified into 11 genome types. These genotypes include six diploids (AA, BB, CC, EE, FF, and GG), and five tetraploids (BBCC, CCDD, HHJJ, HHKK, and KKLL) [4,5]. Within the collection of wild rice species in the genus *Oryza*, *O. granulata* belongs to the *O. meyeriana* complex that characteristically possesses the GG genome and falls within the lower cladogram in the *Oryza* phylogeny [4,6]. The wild rice genomes serve as a valuable reservoir of genetic data, offering crucial information for investigating the genome evolution [7]. While the occurrence of severe droughts significantly underscored the genetic engineering of drought-tolerant rice cultivars. The absence of adequate knowledge on drought-tolerant species within the genus *Oryza* to facilitate the breeding of highland rice represents an enormous challenge because the optimum growth and output of most species within the genus *Oryza* inherently ocurrs under damp and even aquatic conditions. *O. granulata* is a resilient shade- and drought-tolerant upland wild rice species and poses durable resistance to economically essential pests and pathogens compared to other cultivated and non-cultivated rice species [8]. Genomic and bioinformatic advancements have significantly propelled rapid genomics, transcriptomics, and metabolomic investigations on *Oryza* species [9,10,11,12,13,14], setting the scientific groundwork for future improvements in *Oryza* characteristics and assuring global rice safety. However, human disturbance and habitat fragmentation made many natural populations of *O. granulata* endangered and some even went extinct [15]. Although our previous studies have obtained novel insights into the population genetic structure of this wild species [16,17], efficient conservation strategies are urgently required to sustain the quality and integrity of the gene pool.

The nuclear, mitochondrial (mitogenome), and plastid genomes are the three relatively independent genetic components typically found in a plant cell. Mitochondria are double-membraneous and ubiquitous cellular organelles that are the primary sites for oxidative metabolism and energy transformation in eukaryotic cells, and are often referred to as the “powerhouse” or “energy factory” of cells [18]. As semi-autonomous organelles, mitochondria have a genetic architecture independent of the nucleus [19]. Plant mitogenomes have become crucial tools for efficient classification, determining the origin of species, and gaining insights into the phylogeny [20,21,22]. Generally, prevailing variations in genome size, the spatial distribution of genes, the proportion of non-coding DNA sequences, the abundance of repetitive DNA sequences, the capacity to incorporate foreign DNA, the proportion of conserved gene sequences, and the proportion of RNA-editable genes and unique genomic features are frequently deployed to distinguish between genomes. Typically, distinguishing features of *Oryza* mitogenomes are common in the mitogenomes of land plants [23,24,25,26]. Recent studies have demonstrated mitochondria’s unique and significant roles in promoting plant growth and development [27,28]. These studies also revealed that mitochondria are strongly associated with agronomic qualities such as cytoplasmic male sterility, disease resistance, and plant growth vigor [29,30]. Compared to chloroplast genomes, there is limited research on the intricate structure dynamics of plant mitogenomes. Besides the nuclear [31,32] and chloroplast [33] genomes, there are no reports on *O*. *granulata* mitogenomes in public repositories, including the NCBI GenBank database. Efforts are needed to generate the mitogenomic data of *O. granulata* in order to comprehend how it evolved and how to conserve and utilize its precious genetic diversity.

In the present study, we integrated data from third-generation PacBio sequencing and second-generation Illumina sequencing of *O. granulata*. We assembled and annotated the full *O. granulata* mitogenome. Additionally, we examined the gene content, genome structure, and evolutionary phylogeny of O. granulata with other species in the genus *Oryza*. We performed a comparative mitogenome analysis of *Oryza* species to obtain vital genome information, including variable genomic regions, conserved regions, and incidence of mitogenome reshuffling or rearrangements. Additionally, we looked into the transfer of genes between *O. granulata’s* chloroplasts and mitogenomes. Such a mitogenome contains information that can support the development of efficient molecular markers, carry out genetic engineering, and explain the phylogenetic and evolutionary relationships among the *Oryza* species.

## 2. Materials and Methods

### 2.1. Plant Material Collection and Genome Sequencing

Genome sequence data were generated using DNA extracted from dozens of *O. granulata* plants sampled from Menghai County, Yunnan Province, China. Briefly, seeds harvested from *O. granulata* plant growing in Menghai County, Yunnan, were planted in the greenhouse at Kunming Institute of Botany, Chinese Academy of Sciences. Fresh, healthy, and intact leaves harvested from the individual seedlings were immediately frozen in liquid nitrogen and later transferred into a −80 °C laboratory freezer before DNA extraction. Total DNA was extracted using a modified CTAB method [34]. NanoDrop 2000 spectrophotometer (NanoDrop Technologies, Wilmington, DE, USA) and electrophoresis on a 0.8% agarose gel were deployed for qualitative and quantitative assessments of the extracted DNA, respectively. PacBio SMRT and Illumina Hiseq sequencing platforms were deployed to generate high-quality third-generation and second-generation genome sequencing data.

### 2.2. Mitochondrial Genome Assembly and Annotation

Raw reads generated with PacBio SMRT technology were mapped to the complete *Oryza sativa* L. spp. *japonica* cv. Nipponbare (Nip) mitogenome (Accession Number: NC011033) [35] using BWA v0.7.17 [36] and SAMtools v1.12 [37]. The mitochondrial genome was generated through a complete de novo assembly using CANU v1.8 [38] using the automatic pipeline with default parameter settings. PacBio-associated sequencing errors in the final assembly were resolved using Pilon v1.23 [39] (available at https://github.com/broadinstitute/pilon, (accessed on 8 June 2023)) using Illumina data with the default parameters. Candidate mitochondrial genome contigs were identified by a BLASTn search [40] and further verified through Sanger sequencing.

The mitochondrial genome was manually annotated with Mitofy, an online homology-based prediction tool [41] (http://dogma.ccbb.utexas.edu/mitofy/, (accessed on 12 June 2023)). The tRNA and rRNA were annotated using tRNAscan-SE v1.21 [42] and RNAmmer v1.2 [43]. Genome maps were drawn with OGDRAW [44] and edited with the Adobe Illustrator CS6.

### 2.3. SSRs and Repeat Sequences 

The simple sequence repeats (SSRs) in the assembled mitogenome were identified using a Perl script MISA [45]. The minimum number of mononucleotide (mono-) repeats, dinucleotide (di-) repeats, trinucleotide (tri-) repeats, tetranucleotide (tetra-) repeats, pentanucleotide (penta-) repeats, and hexanucleotide (hexa-) repeats were set as 8, 4, 4, 3, 3, and 3, respectively. Furthermore, forward, reverse, palindromic, and complementary repeat sequences were identified using the Vmatch [46] repfind.pl program, which replaced the online website REPuter [47] with the following settings: a hamming distance of 3 and a minimal repeat size of 30 bp.

### 2.4. Prediction of RNA Editing Sites

The prediction of C to U RNA editing sites in the mitogenome PCGs was performed using the Deepred-mt [48]. The Deepred-mt tool’s prediction module involves convolutional neural network (CNN) model predictions with high accuracy compared to previous prediction tools. Results with probability values above 0.6 were chosen.

### 2.5. Mitochondrial Plastid Sequences and Collinearity Analysis

*O. granulata* chloroplast genome sequence was retrieved from the NCBI GenBank (Accession Number: KF359920) [33], and BLAST v2.11.0+ [40] was used to identify comparable sequences in the mitochondrial and chloroplast genomes. The circus plot was drawn using TBtools [49] by calling the Advanced Circos program [50]. 

We selected two additional species, *O. rufipogon* (RUF) and *O. sativa* L. spp. *japonica* cv. Nipponbare (Nip) (Table S6) to conduct a colinear analysis with *O*. *granulata*. Colinear blocks were identified based on sequence similarity using the BLASTn v2.11.0+ [40] program, employing the e-value parameter 1 × 10^−5^. The multiple synteny plot was drawn using LINKVIEW2 v1.0.5 (https://github.com/YangJianshun/LINKVIEW2/, (accessed on 12 July 2023)).

### 2.6. Phylogenetic Analysis

The published mitogenomes of fourteen plant species (*O. rufipogon*, *O. sativa* L. spp. japonica Nipponbare, *O. sativa* L. spp. indica 9311, *O. coarctata*, *O. minuta*, *Zea luxurians*, *Zea mays*, *Sorghum bicolor*, *Eleusine indica*, *Triticum aestivum*, *Phoenix dactylifera*, *Glycine max*, *Vitis vinifera*, and *Arabidopsis thaliana*) were downloaded from the NCBI database and used for phylogenetic analysis with Ginkgo biloba selected as the outgroup (Table S6). In all, fourteen conserved protein-coding genes, including atp9, ccmC, ccmFn, cob, cox1, cox2, cox3, matR, nad3, nad4L, nad6, nad9, rps12, and rps4, were identified and used for subsequent phylogenetic analyses. The MAFFT v7.505 program [51] with default parameters was used to protein sequences. Sequences were aligned end-to-end and trimmed using Gblocks v0.91b [52] with the default parameters. The trimAl v1.4. rev15 [53] was used for aligning and converting sequences from fasta to nexus format. The Markov Chain Monte Carlo (MCMC) in version 3.2.7a of MrBayes was used in the iterative analysis method [54] to obtain a simulation pattern for a population of 500,000 generations with samples taken at intervals of 100 generations, and produced a high-frequency tree. The quality of the phylogenetic tree generated was further polished with the online website ITOL v6.8 [55].

## 3. Results

### 3.1. Structural Characteristics of the O. Granulata Mitogenome

**Figure 1:**
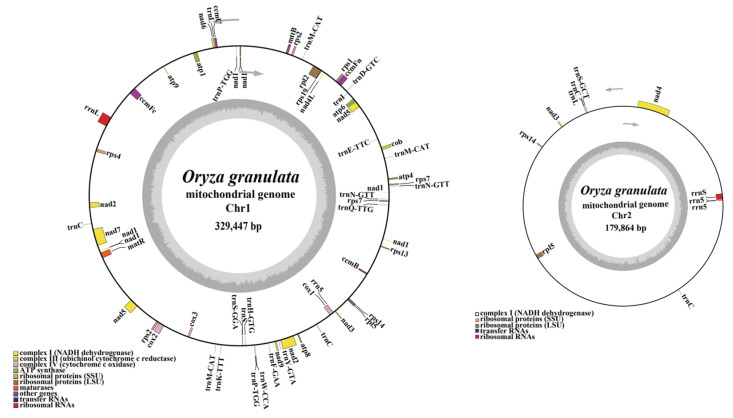
Graphical maps of Chr1 and Chr2 in the *O. granulata* mitogenome. The inner circle in gray denotes the GC content of the two individual chromosomes. The different color codes distinguish genes belonging to diverse functional groups. Gene transcripts clockwise or counter-clockwise strands are drawn on the inside and outside of the circles, respectively.

To obtain a complete mitogenome for *O. granulata* through the integrated assembly of Illumina and PacBio reads. Firstly, we filtered mitochondrial sequence reads from whole genome PacBio sequencing data of *O. granulata*. About 4.3 Gb of the filtered *O. granulata* mitochondria sequence reads aligned to the previously reported mitogenome of Nip [35] and covers an average-sized *Oryza* mitogenome (450~550 kb) [56] over 8600 times. We obtained the two complete circular chromosomes, Chr1 and Chr2 (Figure 1), and a total mitogenome length of 509,311 bp. The obtained *O. granulata* mitogenome’s features are similar to features previously reported for *Oryza* mitogenome, especially for Nip [35], RUF [57], and *O. minuta* [58]. The two chromosomes of the mitogenome had a length of 329,447 bp and 179,864 bp and total GC contents of 43.70% and 43.19%, respectively (Figure 1). 

The *O. granulata* mitogenome encoded thirty-seven protein genes, five rRNA genes, and twenty-five tRNA genes (Table 1), totaling fifty-six unique genes (sixty-seven including repeats). *Nad3*, *rpl5*, *rps7*, *rps14*, *trnN-GTT*, *trnM-CAT*, and *trnP-TGG* all had two duplicated copies, *rrn5* had three duplicated copies, and *trnC* had four duplicated copies. Intergenic spacers constituted the largest part (468,954 bp, 92.08%) of the *O. granulata* mitogenome, and protein-coding sequences comprised only 6.42% (32,679 bp) of the total length. The non-coding sequences of the *O. granulata* mitogenome were almost 93.58%, which is higher than the average of (89.46%) the coding sequences previously reported for angiosperm [59].

### 3.2. Repeat Sequences

**Figure 2:**
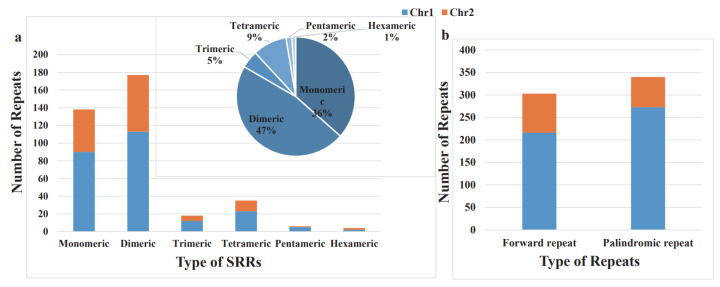
The simple sequence repeats (SSRs) and dispersed repeats identified in the *O. granulata* mitogenome. (**a**) Type and number of SSRs. The blue and orange legends indicate Chr1 and Chr2 of the *O. granulata* mitogenome; (**b**) Dispersed repeats (≥30 bp, distributed within the same chromosome) identified on the two chromosomes.

Simple sequence repeats (SSRs) are units of sequence repetition that can be one to six base pairs long [60]. Due to their polymorphism, simplicity in PCR detection, co-dominant inheritance, and widespread genome coverage, SSRs are helpful [61]. Simple sequence repeats (SSRs) assessment results revealed 378 SSRs across the two chromosomes in the *O. granulata* mitogenome, with dimeric repeats being the most abundant (Figure 2), while monomeric SSRs accounted for 36%. Of these SSRs, monomeric thymine (A/T) repeats occupied 92.03% (127/138). Dimeric SSRs occupied 47% of the total SSRs, and AG/CT repeats accounted for 54.24% (96/177) of dimeric SSRs. Besides the major types of SSRs, we also identified eighteen trimeric SSRs, thirty-five tetrameric SSRs, six pentameric SSRs, and four hexametric SSRs (Table S1). 

Additionally, 643 pairs of dispersed repeats with minimum lengths of 30 bp were identified in the two chromosomes of the *O. granulata* mitogenome. These dispersed repeats comprised 303 pairs of forward repeats, 340 pairs of palindromic repeats, and 340 palindromic repeats; the longest palindromic repeat of 3100 bp was recorded as the length of the longest forward repeat (Figure 2 and Table S2). 

### 3.3. Prediction of RNA Editing Sites

**Figure 3:**
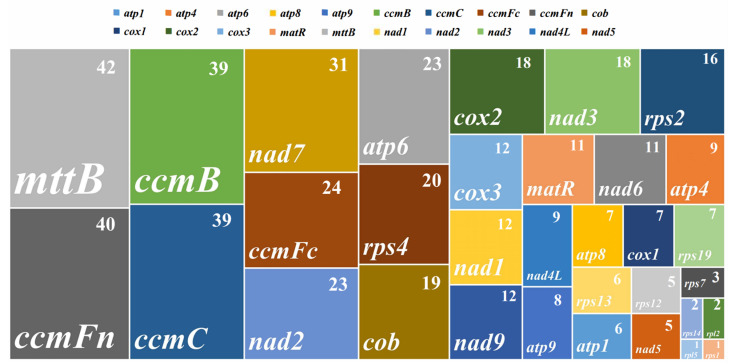
The distribution of RNA editing sites across the protein-coding genes of the *O. granulata* mitogenome. Numbers in the upper right corner of the rectangle denote numbers of RNA editing sites on each protein-coding gene, and colors represent various protein-coding genes.

Except for mosses, RNA editing events, including the insertion, deletion, or substitution of nucleotides, are restricted to conserved RNA-coding regions in terrestrial plants [62,63]. Deepred-mt aided analysis revealed 488 potential C to U RNA-editable sites in 33 mitochondrial protein-coding genes (Figure 3). At the same time, we predicted the RNA-editable sites for the individual genes (Figure 3 and Table S3). Among these mitochondrial genes, *mttB* genes have 42 RNA-editable sites, while 40 RNA-editable sites were identified in *ccmFn*. The *mttB* and *ccmFn* gene groups contain the top protein-coding genes. Additionally, we observed that individually, *ccmC*, *ccmB*, and *nad7* contain more than 30 RNA-editable sites. Meanwhile, C to U were the only RNA-prone editing sites identified in the rpl5 and *rps1* gene groups. 

### 3.4. Homologous Sequence Analysis of Mitochondrial and Plastid Genomes

**Figure 4:**
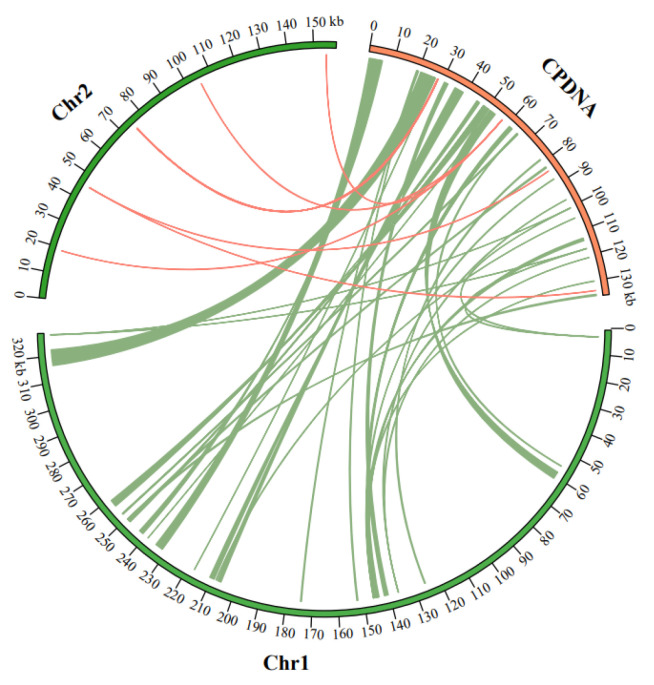
The sequence transfer between the two mitochondrial chromosomes and chloroplast genome of *O. granulata*. The two chromosomes (Chr1 and Chr2; green) of the mitogenome and the chloroplast genome (CPDNA; orange) of *O. granulata* are depicted with a circular diagram. The linkage indicates the shared transfer sequence between the mitogenome and the chloroplast genome.

Higher plants’ mitogenomes contain substantial sequences that have migrated from their plastomes and nuclear genomes. This study identified 57 MTPTs between the *O*. *granulata* mitogenome and chloroplast genome, with a sequence similarity greater than 80% in each matching pair. These 57 MTPTs recorded a length of 30,349 bp, thus constituting 5.96% and 22.32% of the mitogenome and chloroplast genome, respectively. The individual length of the MTPTs MTPT1, MTPT8, MTPT9, MTPT10, MTPT11, MTPT17, and MTPT19 exceeded 1000 bp in length with MTPT1 being the longest, spanning 6166 bp. MTPT48 and MTPT52 had the shortest length of 30 bp (Figure 4; Table S4). 

### 3.5. Comparative Mitochondrial Genomic Analyses of the Oryza Species

**Figure 5:**
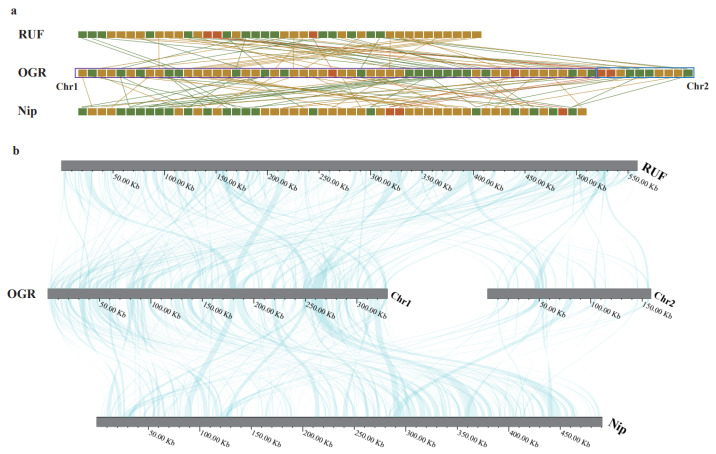
Evidence for mitochondrial rearrangement and mitogenome synteny in the genus *Oryza*. (*O*. *granulata*, OGR; *O*. *rufipogon*, RUF; *O. sativa* L. spp. *japonica* cv. Nipponbare, Nip) (**a**) The linear order of genes (orange, rRNA; green, tRNA; yellow, protein-coding genes) in the mitochondrial *O. granulata* genomes (Chr1, purple box; Chr2, blue box) and related plant species. Genes with the same annotation are connected with lines. Boxes are not proportional to actual gene length; (**b**) Bars indicate the mitogenome, and the ribbons denote the homologous sequences between the adjacent species.

The mitogenomes of RUF and Nip were compared with *O. granulata* to assess mitogenomic rearrangement and collinearity. Despite minimal variances in gene content, there were significant changes in gene order among the sequenced *Oryza* mitogenomes (Table 1 and Figure 5a). Similarly, minimal variation occurred at the nucleotide level (Figure 5b). However, the high incidence of rearrangements caused low levels of collinearity between mitogenomic sequences from *O. granulata* and the other two *Oryza* species, with only a few of these rearrangements rearranging significant DNA sequences within *O. granulata*. Findings from these investigations showed that while many homologous collinear blocks were detectable in *O. granulata* and the other two *Oryza* species, the lengths of these blocks were relatively short, indicating a high degree of non-conservation in the mitogenome sequences (Figure 5b and Table S5). Furthermore, the arrangement trends displayed by the collinear blocks in the *Oryza* mitogenomes are unstable, primarily due to the frequent incidence of genome reorganization in *O. granulata* and related species. Additionally, we found numerous regions lacking homology between these mitogenomes.

### 3.6. Phylogenetic Analysis

**Figure 6:**
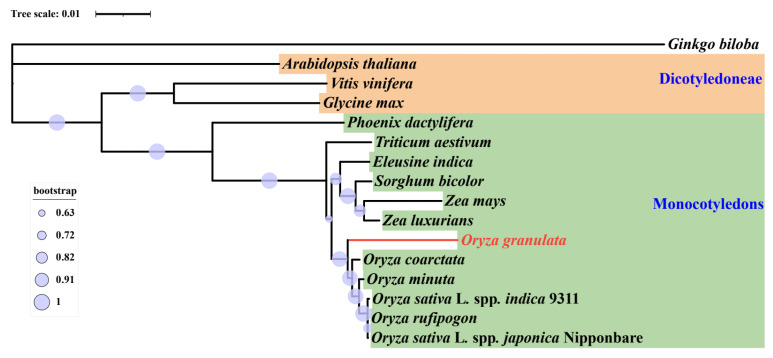
The phylogenetic relationships of *O. granulata*. The Bayesian tree was constructed based on the 14 genes common in the 16 plant mitogenomes. *Ginkgo biloba* was set as an outgroup. The purple circle represents bootstrap support values.

Mitogenome-based evolution among the examined species in the genus *Oryza* was investigated through the deployment of protein sequences of conserved protein-coding genes from *O. granulata,* and fifteen plant species were deployed for phylogenetic analyses and obtained a well-supported phylogenetic tree, with most of the nodes having Bayesian bootstrap values > 90%, showing the reliability of the recovered phylogeny (Figure 6). The results revealed that the investigated species formed the two major clusters, monocotyledons and *dicotyledons* with *G. biloba* as an outgroup. Furthermore, we found that the clustering matches the individual species, their corresponding families, and their genera, confirming the reliability of mitogenome-dependent clustering. The phylogenetic tree indicated that *O. granulata*, *O. coarctata*, *O. minuta*, *O. sativa* L. spp. *indica* 9311, *O. sativa* L. spp. *japonica* Nipponbare, and *O. rufipogon* were grouped together, and *O. granulata* was located at the base of the *Oryza* phylogeny. 

## 4. Discussion

Plants receive life-sustaining energy from mitochondria and possess larger and more sophisticated mitogenomes than animals due to the abundance of repetitive sequences [64]. Numerous studies have established that the expansion of the angiosperm mitogenome is due to rapid buildups in repetitive sequences and the frequent insertion of foreign sequences in the cause of foreign sequences via horizontal transfer [65,66]. The morphological architecture of mitochondria in angiosperms assumes diverse conformations. For instance, under different recombination events, mitochondria can be linear, circular, extremely branching, sigma-like, or networked [20]. The *O. granulata* mitogenome-assembled resource generated in this study contains two circular structures that are similar to the core structure of the mitochondrial genome of MingHui63 (*Oryza sativa* L. spp. *indica* cv. MingHui63) [67]. The length of the *O*. *granulata* mitogenome was 509,311 bp, and its genome size is somewhat close to other *Oryza* species, such as Nip (490,520 bp) [35], Shuhui498 (527,116 bp) [68], *O. coarctata* (491,065 bp) [69,70], and *O. minuta* (515,022 bp) [58], but differ significantly from *Camellia sinensis* (800~1200 kb) [71,72], *Arabidopsis thaliana* (367,808 bp) [73,74], and *Ginkgo biloba* (346,544 bp) [75]. The GC content of the *O. granulata* mitogenome was 43.52% and fell in line with the GC content reported for the above species, indicating tight conservation in GC content during evolutionary course of angiosperms.

Generally, there is no positive correlation between gene numbers and the proportion of protein-coding genes identified in plant mitogenome due to the high incidence of gene loss and accumulation of multiple gene copies resulting from the rearrangements of repeat sequences [76,77,78]. We annotated thirty-seven genes coding proteins, twenty-five genes coding tRNA, and five rRNA-coding genes in the assembled mitogenome of *O. granulata*. Besides these protein-coding genes, tRNA and rRNA genes identified in the *O. granulata* mitogenome were also in multiple copies. In contrast, the *O. granulata* mitogenome has lost *rpl10*, *rpl16*, *rps3*, *rps10*, *rps11*, *rps12*, *sdh3*, and *sdh4* genes. Before being lost from the mitogenome, we speculated that a significant proportion of these genes, if not all, may have functionally moved into the nucleus [35]. These two pronounced patterns, and, thus, the significant reductions in the proportion of lineage-specific protein-coding genes recorded as mitochondrial, and the seemingly mitogenomic restrictive loss of all ribosomal protein and *sdh* genes are in tandem with the gene loss pattern observed in other species within the genus *Oryza* [75].

The plant mitogenome contains many repeat sequences which the SSRs have extensively used as bio-markers to identify and assess genetic variation in species [79]. Characteristically, all monomeric SSRs in the *O. granulata* mitogenome consisted of A or T nucleotides, making mitogenomes richer in A/T content than G/C content. Multiple research findings suggested that plant mitogenomes are rich in A/T content [80,81]. The significant enrichment of the *O. granulata* mitogenome in AT repeats likely accounted for the high A/T content. The current investigations showed that the predominant proportion of 515 pairs (80.09%) of the dispersed repeat sequences identified in the mitogenome of *O. granulata* ranged between 30–100 bp, which is comparatively higher than the proportion of 30–100 bp types of dispersed repeat sequences reported in many plants. Plant mitogenomes are not a single circle but a complex and dynamic variety of shapes [82]. In plant mitogenomes, repetitions frequently mediate homologous recombination locations. Numerous large repeats may serve as intra- or intermolecular recombination, generating various configurations or isoforms [83,84]. Additionally, recombination caused by lengthy repeats occurs more frequently than short repetitions. It is also possible that the splitting of more substantial circular chromosomes produced the numerous chromosomes of the *O. granulata* mitogenome.

The mitogenomes of higher plants contain large amounts of RNA-editable genes and have been shown to play core roles in the initiation of essential steps in the expression of genes [85]. It belongs to the class of alterations made after transcription. Typically, the progression in angiosperms proceeds through site-specific cytosine (C) conversion to uracil (U). RNA editing events accelerate the evolution of homologous mitochondrial proteins across species [86]. These start and stop codons typically mediate the emergence of more conserved and homologous proteins between species, allowing for improved gene expression in mitochondrial counterparts in other species and improving gene expression in mitochondria [87]. RNA editing processes have been cited as a crucial regulatory process in plant development and growth [88,89,90]. We discovered 488 RNA editing sites in the *O. granulata* mitogenome, which is remarkably similar to those in other terrestrial plants [25,26,91]. Meanwhile, the *rpl5* and *rps1* genes only have one RNA editing site, indicating that the *rpl5* and *rps1* genes are conserved.

Dynamic intercellular phenomenons, including horizontal gene transfer (HGT), and intracellular gene transfer (IGT), are vital fundamental parameters that are known to drive the exchange and the integration of organellar and nuclear genomes and DNA fragments into nuclear genomes to create NUMTs and NUPTs in plants [92,93]. Studies have shown that plastome to mitogenome often undergo frequent orientation, and re-orientation occurs between the two plant organellar genomes. We observed massive mitochondrial plastid fragments (MTPTs) in the plant mitogenomes. Plant mitogenomes typically contain 0.56 percent (*M. polymorpha*) to 10.85 percent (*P. dactylifera*) of sequences originating from plastids [94]. Here, we similarly detected MTPTs in the *O. granulata* organellar genomes and found that the *O. granulata* mitogenome integrated 57 (30,349 bp) MTPTs. In angiosperm, tRNA-coding genes are often translocated from the chloroplast genome to the mitochondrial genome [76].

Mitogenomic collinearity results showed that sequences of the three *Oryza* species are non-conservative, indicating the likely occurrence of rapid genome recombination throughout the lengthy evolutionary process. Contrary to the outstanding level of conservation seen in animal mitogenomes [95,96], significant rearrangement has been observed in the mitochondrial genomes of several plant families [58]. Therefore, rearrangements at the whole-genome level are possibly a significant driving force for the evolution and mitogenomic diversification of *Oryza*.

The phylogenetic tree inferred based on six *Oryza* species and ten other plant species’ mitochondrial genomes was consistent with the taxonomic data associated with those species. These results demonstrate the potential for using data from organelle genomes in plant phylogeny research [97]. The availability of the *O. granulata* mitogenome and comparative genomic analysis provides a solid foundation for future *Oryza* plant-relatedness studies. Furthermore, the current study will serve as a valuable alternative methodology for investigating plant diversity and evolution.

## Figures and Tables

**Table 1:** Gene composition of the *O. granulata* mitogenome.

Group of Genes	Genes	
	Chr1	Chr2
Complex Ⅰ NADH dehydrogenase	*nad1*, *nad5*, *nad4L*, *Nad6*, *nad2*, *nad7*, *nad9*, *nad3*	*nad4*, *nad3*
Complex Ⅲ Cytochrome c biogenesis	*cob*	
Complex Ⅳ Cytochrome c oxidase	*cox2*, *cox3*, *cox1*	
Complex Ⅴ ATP synthase	*atp4*, *atp6*, *atp1*, *atp9*, *atp8*	
Ubiquinol cytochrome c biogenesis	*ccmFn*, *ccmC*, *ccmFc*, *ccmB*	
Ribosome large subunit	*rpl2*, *rpl5*	*rpl5*
Ribosome small subunit	rps 7*2, *rps1*, *rps19*, *rps2*, *rps4*, *rps14*, *rps13*	*rps14*
Ribosome RNA	*rrnL*, *rrn5*	*rrnS*, rrn5*2
Transfer RNA	trnN-GTT*2, trnM-CAT*2, *trnE-TTC*, *trnI*, *trnD-GTC*, trnP-TGG*2, *trnL*, trnC*2, *trnM-CAT*, *trnK-TTT*, *trnS*, *trnS-GGA*, *trnH-GTG*, *trnW-CCA*, *trnF-GAA*, *trnY-GTA*, *trnQ-TTG*	trnC*2, *trnL*, *trnS-GCT*
Maturases	*matR*	
Transport membrane protein	*mttB*	

Note: The number following the * denotes the copy number in the genome.

## Data Availability

The genome assembly has been added to the GenBank database of the National Center for Biotechnology Information (NCBI) (Accesssion Numbers: OR539813 and OR539814; available online at http://www.ncbi.nlm.nih.gov/genbank, (accessed on 11 October 2023)).

## Supplementary Materials

The following supporting information can be downloaded at: https://www.mdpi.com/article/10.3390/life13112114/s1, Table S1: Frequency of simple sequence repeat (SSR) types in the *O. granulata* mitogenome; Table S2: Long repeats (repeat unit ≥ 30 bp) in the *O. granulata* mitogenome; Table S3: The predicted C to U RNA editing sites in mitochondrial protein-coding genes of *O. granulata*; Table S4: The homologous DNA fragment identified among the mitogenome and chloroplast genomes of *O. granulata*; Table S5: Sequence collinearity of the mitogenome of *O. granulata* and related plant species; and Table S6: The plant species used for phylogenetic analysis.
